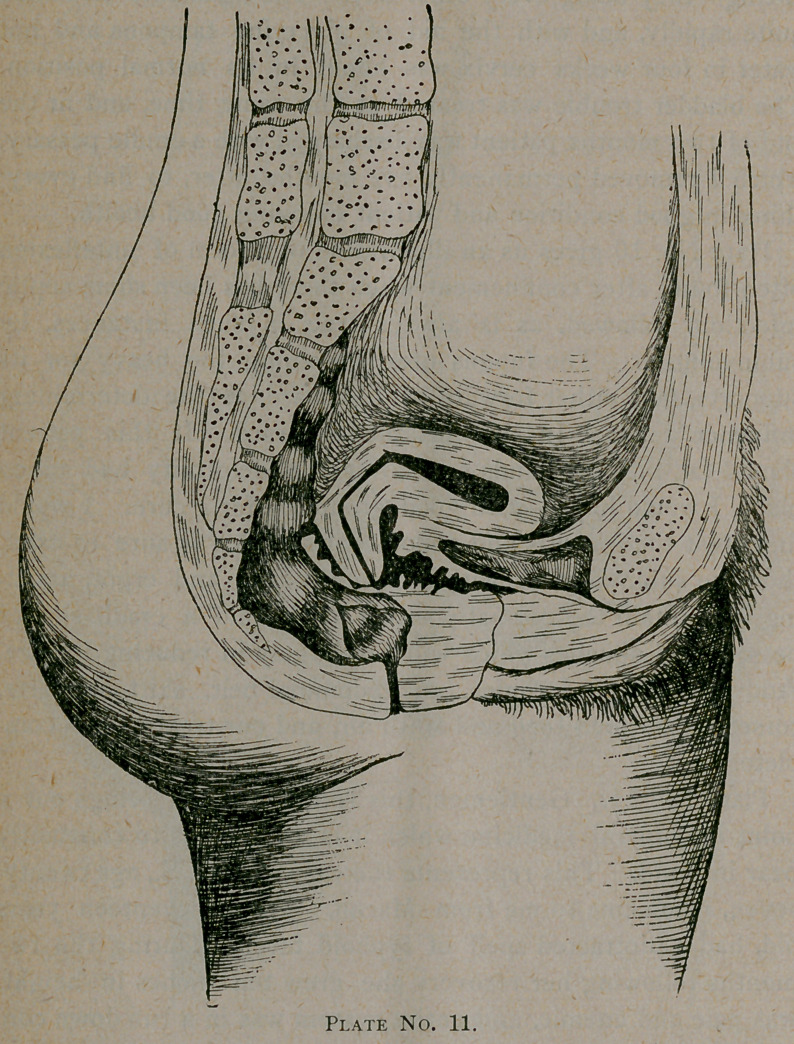# Ante and Retro—Positions of the Uterus—Their Pathology, Symtomatoly and Treatment

**Published:** 1893-09

**Authors:** W. W. Stewart

**Affiliations:** Columbus, Ga.


### Southern Medical Record.

A MONTHLY JOURNAL OF MEDICINE AND SURGERY.

VOL. XXIII. ATLANTA, GA., SEPTEMBER, 1893. No. 9

### Original Articles.

### ANTE AND RETRO-POSITIONS OF THE UTERUS—THEIR PATHOLOGY, SYMTOMATOLOGY AND TREATMENT.*

BY W. W. STEWART, M. D., COLUMBUS, GA.

Our next case, illustrated in plate No. 6, is another form of retroversion which for its causation has a contraction of the sacro-uterine ligaments, without other pathological conditions than those secondary to version.

The bladder in all of these cases as you can see is almost surely involved.

In this case the contractions were so firm that at first it similated adhesion. It was with great effort the uterus was restored to the normal position and a long course of tamponing was required, the cul-de-sac being well packed before the ligaments were sufficiently stretched to allow the use of a pessary, which patient has now worn for seven months.

It was removed two weeks ago and uterus remains now in normal position.

How long it will remain so is more than I can say.

Plate No. 7. represents another case which is in striking contrast with No. 6, the pathological lesion being the same. Here we have elevation of the uterus with anteflexion of the

* Continued from page 399.

cervix. This is a rather hopeless condition. The ligaments are hard to stretch on account of the cul-de-sac being obliterated and taken up by the flexion of the cervix.

PLATE No. 6.

The best treatment in this instance is to open the cervical canal by incision of the lower lip of the cervix and the removal of a part of the angle of the upper portion at the internal os, and subsequent use of a glass flanged tube or stem pessary.

The former is preferable, to be worn for six or eight months and dilation of the canal practiced at intervals of two months for a year thereafter.

PLATE NO. 7.

In plate No. 8 we have the same uterine deformity; but in this case the pathology is markedly different.

We have no inflammatory condition of the ligaments in this instance, the deformity here being caused by impaired nutrition, constipation and tight lacing.

In this case we can hope for good results. We will first remove all weight and tight bands from the waist by supporting the clothing from the shoulders. Second, correct the constipation. Third, introduce a Schultz figure of 8 pessary which, in

PLATE No. 8.

pushing the cervix backward, will correct the existing anteflexion of the cervix. Add to this good exercise and fresh air and a cure will promptly reward your efforts, provided the trouble has not existed so long that marked degenerative changes have taken place at the point of flexion.

Plate No. 9 represents an interesting case sent me from Eufaula, Alabama. Mrs. R., age twenty-five, had two children.

PLATE NO. 9.

With the first there was no trouble, patient being perfectly well till the birth of second child, two years ago, at which time she had some slight septic trouble which caused a rise of temperature for several days, which soon subsided. She had at this time some soreness over abdomen with tympanites. On coming to me her greatest complaint was from bladder in the form of chronic cystitis, and also obstinate constipation, with chronic dyspepsia and meteriorism. On examination a clear

PLATE NO. 10.

diagnosis was made of anteversion, due to chronic posterior parametritis and metritis. The sacro-uterine ligaments were contracted, and cervix fixed. Bladder was examined with cysto-scope and a large ulcer detected, caused by decomposition of urine in the bladder. Treatment consisted, first, in curetting and packing uterus with iodoform gauze. After four days this was withdrawn and a tenaculum caught in posterior lip of cervix, and gentle traction practiced for ten minutes at each sitting—they being every other day. The ligaments stretched quite rapidly, and with the aid of glycerine tampons and hot water in four weeks cervix was about in its normal position. The bladder trouble was treated at the same time, and at the end of two months patient was discharged with a cradle pessary, which I removed permanently five months after, to find everything in good condition and patient enjoying good health.

Plate No. 10 gives us an illustration of a form of anteflexion often found after confinement when there has been some septic infection, followed, as is always true in such instances, by subinvolution. The fundus uteri becoming too heavy for its supports, falls either forward or backward, as the determining influence tends. In this instance forward. If this patient should fall into my hands soon after the deformity had taken place, I would hope—with good grounds—for a cure. I would first treat the metritis and endometritis, which is sure to exist, then the subinvolution, with the potash salts and ergot, assisting it to retain the normal position by the aid a cradle-shaped pessary and douches which will aid largely in reducing its size. Add to this good tonics, an abdominal belt, skirt supports, corset waist, good food and fresh air, and our treatment is complete.

Plate No. 11. Gentlemen, this is a case which brings out a point in general medicine which it is well for us to constantly bear in mind. This represents the case of Mrs. S., age twenty-seven, who came to me from Macon. When seventeen years old, had a protracted spell of typhoid fever. During the two months following her recovery she grew ten inches in height, was pale and anemic, and entire system was in a run down condition. Being a young lady of fashion, she was put into corsets at once. Three months after her recovery, she menstruated for the first time after her illness. For two days prior, and all during its course, she suffered intense pain, bearing down in character. This dysmenorrhœa continued, gradually increasing. Appetite became capricious, bowels constipated, and the long line of symptoms mentioned in the first of this paper developed. At twenty-two she married, after which all symptoms became

PLATE No. 11.

worse until she became what I term a sofa invalid. In this condition she was treated by several physicians in Macon, one in Baltimore, and one in New York.

Then she came into my hands, and the results attained by us all so far are not at all flattering. Her condition now, is as follows: The uterine syndrome is complete; abdomen hyperæsthetic; anteflexion of body of cervix with retro-position and dextro-rotation; bladder irritable; no cystitis; ovaries and tubes exquisitely tender and slightly enlarged. In the last year has developed amenorrhœa, but at each period, though flow consists of about one drachm of bloody mucus, she suffers with a burning, boring pain in uterus, shooting pains and intense headaches. These headaches are more or less continuous during the entire menstrual period. I have both packed and dilated the uterus, and tried to use supports of every conceivable kind, but she can tolerate none of them. Cotton-wool even gives pain. The second flexion is so high that cervical amputation or incision would be of no avail, and ovariotomy is my last resort, which I will perform on my return to Columbus.

The great point of interest in this case is a warning to us all to admonish our patients, convalescing from weakening or wasting diseases, or any condition diminishing muscular tone, to avoid corsets and heavy clothing supended from the waist. Instead of the corset, use the corset-waist. In this case we see the dire results from a disregard of the existing conditions.

## Figures and Tables

**Plate No. 6. f1:**
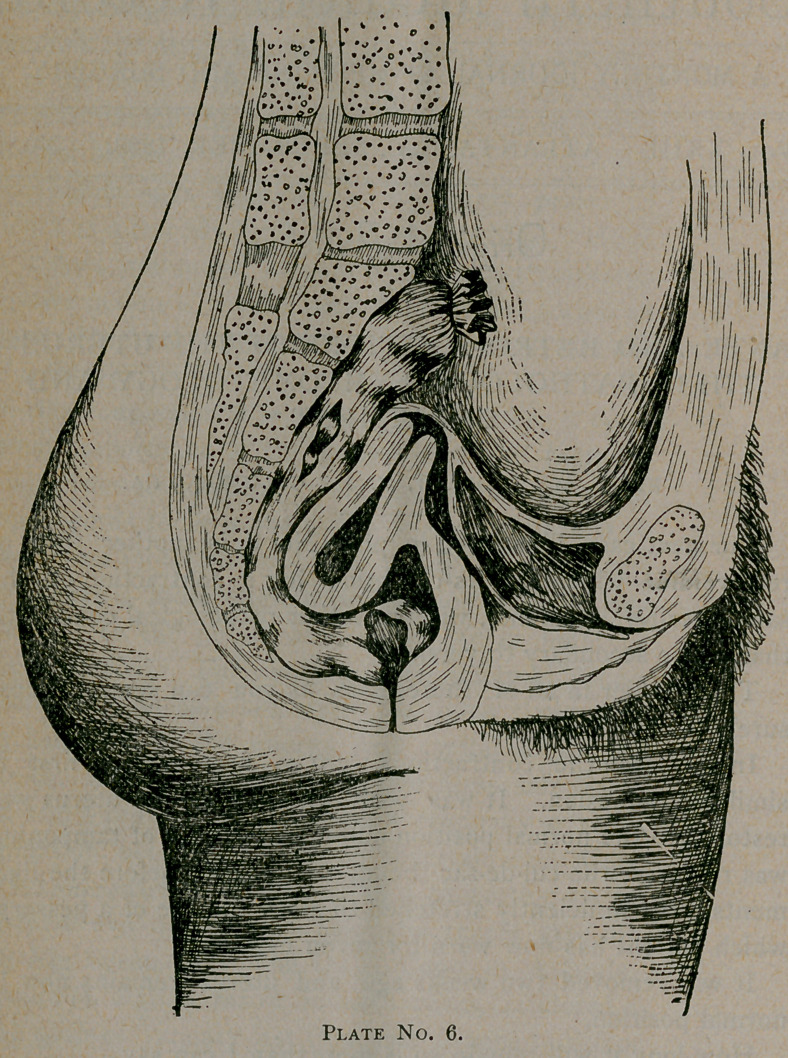


**Plate No. 7. f2:**
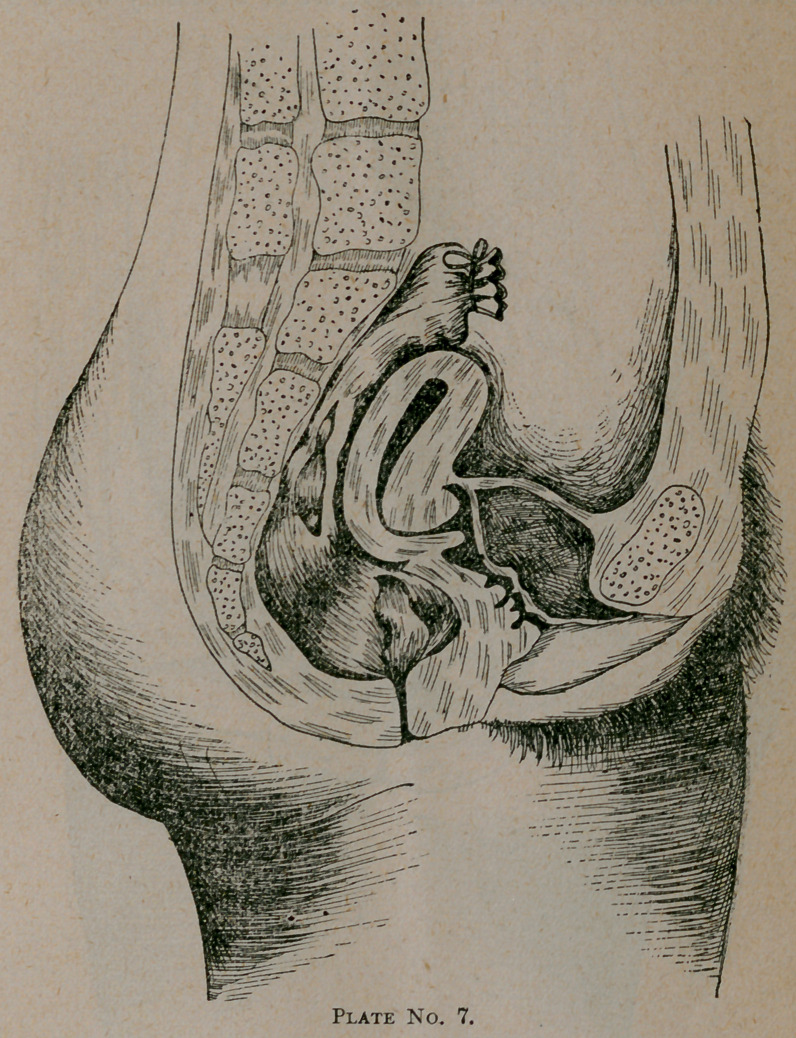


**Plate No. 8. f3:**
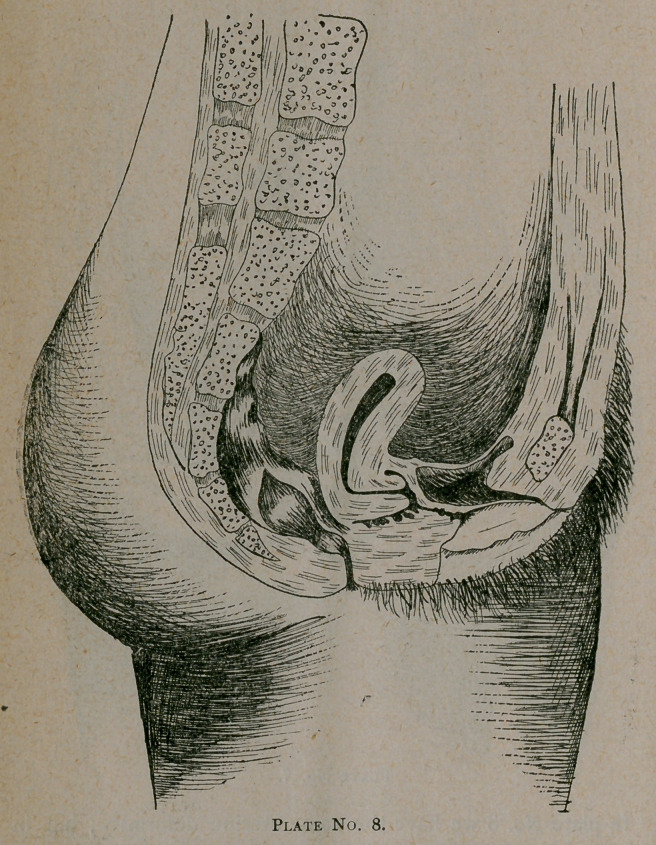


**Plate No. 9. f4:**
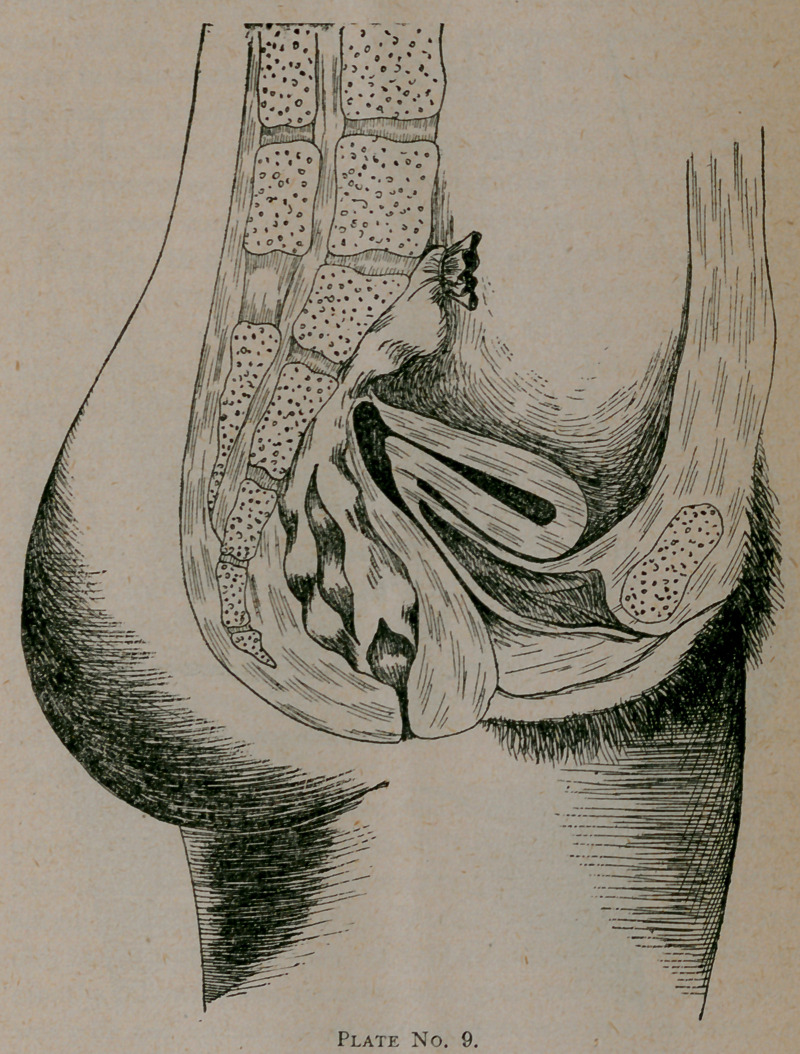


**Plate No. 10. f5:**
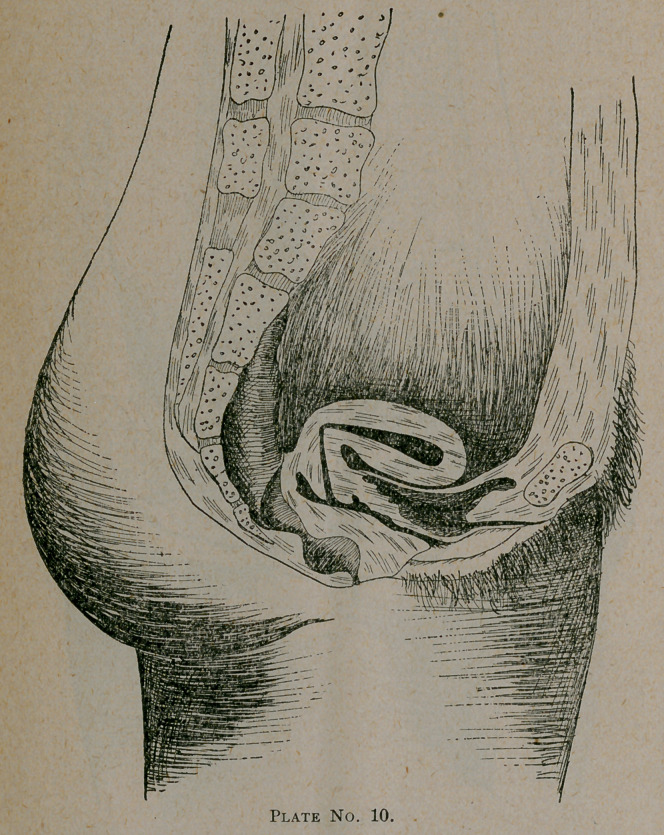


**Plate No. 11. f6:**